# Intravenous Immunoglobulin Prevents Murine Antibody-Mediated Acute Lung Injury at the Level of Neutrophil Reactive Oxygen Species (ROS) Production

**DOI:** 10.1371/journal.pone.0031357

**Published:** 2012-02-17

**Authors:** John W. Semple, Michael Kim, Jing Hou, Mark McVey, Young Jin Lee, Arata Tabuchi, Wolfgang M. Kuebler, Zhong-Wei Chai, Alan H. Lazarus

**Affiliations:** 1 The Toronto Platelet Immunobiology Group, University of Toronto, Toronto, Ontario, Canada; 2 Keenan Research Centre in the Li Ka Shing Knowledge Institute of St. Michael's Hospital, University of Toronto, Toronto, Ontario, Canada; 3 Canadian Blood Services, University of Toronto, Toronto, Ontario, Canada; 4 Department of Pharmacology, University of Toronto, Toronto, Ontario, Canada; 5 Department of Medicine, University of Toronto, Toronto, Ontario, Canada; 6 Department of Laboratory Medicine and Pathobiology, University of Toronto, Toronto, Ontario, Canada; 7 Department of Anesthesia, University of Toronto, Toronto, Ontario, Canada; 8 Department of Surgery, University of Toronto, Toronto, Ontario, Canada; French National Centre for Scientific Research, France

## Abstract

Transfusion-related acute lung injury (TRALI) is a leading cause of transfusion-associated mortality that can occur with any type of transfusion and is thought to be primarily due to donor antibodies activating pulmonary neutrophils in recipients. Recently, a large prospective case controlled clinical study of cardiac surgery patients demonstrated that despite implementation of male donors, a high incidence of TRALI still occurred and suggested a need for additional interventions in susceptible patient populations. To examine if intravenous immunoglobulin (IVIg) may be effective, a murine model of antibody-mediated acute lung injury that approximates human TRALI was examined. When BALB/c mice were injected with the anti-major histocompatibility complex class I antibody 34-1-2s, mild shock (reduced rectal temperature) and respiratory distress (dyspnea) were observed and pre-treatment of the mice with 2 g/kg IVIg completely prevented these symptoms. To determine IVIg's usefulness to affect severe lung damage, SCID mice, previously shown to be hypersensitive to 34-1-2s were used. SCID mice treated with 34-1-2s underwent severe shock, lung damage (increased wet/dry ratios) and 40% mortality within 2 hours. Treatment with 2 g/kg IVIg 18 hours before 34-1-2s administration completely protected the mice from all adverse events. Treatment with IVIg after symptoms began also reduced lung damage and mortality. While the prophylactic IVIg administration did not affect 34-1-2s-induced pulmonary neutrophil accumulation, bone marrow-derived neutrophils from the IVIg-treated mice displayed no spontaneous ROS production nor could they be stimulated in vitro with fMLP or 34-1-2s. These results suggest that IVIg prevents murine antibody-mediated acute lung injury at the level of neutrophil ROS production and thus, alleviating tissue damage.

## Introduction

Transfusion related acute lung injury (TRALI) is currently ranked as one of the most serious complications of blood transfusion today [1], [2]. The majority of TRALI reactions are associated with the presence of anti-HLA and anti-neutrophil antibodies in the transfused products [3]–[6]. It is thought that these leukocyte antibodies primarily stimulate the production of reactive oxygen species (ROS) by pulmonary neutrophils that damages pulmonary vessel endothelium [7]–[12]. Of interest, not all leukocyte antibodies cause TRALI in recipients displaying the cognate antigen [9], [10] and some antibodies e.g. anti-human neutrophil antigen (HNA)-3a and anti-human leukocyte antigen (HLA)-A2 are associated with clinically more severe TRALI reactions [5], [11], [12]. Although the incidence of TRALI is still a matter of debate [13], a recent large prospective clinical study showed that in transfused cardiac surgery patients undergoing a cardiopulmonary bypass procedure, the incidence of TRALI was as high as 2.4 percent of all surgeries [14]. Currently, there is no effective therapy for patients with TRALI reactions except for supportive care such as discontinuation of the transfusion and oxygen therapy.

There have been several animal models of human TRALI including, for example, ex-vivo lung models showing the importance of human anti-neutrophil antibodies in causing lung damage and in vivo models demonstrating how biological response modifiers e.g. lipids and/or platelet-derived CD40L can induce recipient lung damage [15]–[19]. An in vivo murine model of antibody-mediated TRALI was developed in 2006 and has also shown several similarities with human TRALI induction. Looney et al [20] observed that when BALB/c mice were injected with a monoclonal anti-mouse MHC class I antibody (34-1-2s), significant increases in excess lung water, lung vascular permeability and mortality were observed within 2 hours. These adverse reactions were found to be due 34-1-2 s's ability to activate reactive oxygen species (ROS) production by recipient neutrophils in an Fc receptor (FcR)-dependent manner [21]. We subsequently demonstrated that compared with BALB/c mice, mice with severe combined immunodeficiency (SCID) were acutely hypersensitive to 34-1-2 s effects indicating that recipient lymphocytes are important in significantly reducing severe lung damage induced by 34-1-2s [21]. The immunopathologic mechanisms that 34-1-2s utilize have become more complex as Strait et al has recently shown that the antibody induces pulmonary damage by activating macrophages to generate ROS in a complement (C5a)-dependent process [22]. The importance of this latter finding may be that this antibody-mediated model of acute lung injury has at least two immunopathologic events leading to TRALI. Taken together, animal models have been instrumental in better defining the pathophysiology of TRALI reactions.

Immunoglobulin preparations extracted from human blood have been used since the early 1950's to treat immunodeficiency diseases [23], [24]. Intravenous immunoglobulin (IVIg) therapy is also effective in treating bacterial/viral infections and immune regulatory disorders, particularly immunohematologic disorders such as immune thrombocytopenia (ITP) as well as autoimmune neutropenia [25]–[29]. While it's clear beneficial effects in these disorders are well known, its mechanism of action is still debated and several theories have evolved to explain IVIg effects [30]–[39]. To date, prophylactic IVIg administration for antibody-mediated TRALI has not been considered and in fact, there are at least 4 case reports that have demonstrated that IVIg infusions may actually be associated with TRALI reactions [40]–[43]. The mechanism(s) of how IVIg may mediate TRALI in these reports is unknown, however, given IVIg's extensive use in many different immune disorders, these TRALI incidences are quite rare and have not limited IVIg's usage. Since SCID mice, which lack B cell or T cells, were hypersensitive to the effects of 34-1-2s and IVIg has beneficial effects in antibody-mediated disorders, we hypothesized that IVIg may be beneficial for preventing TRALI reactions. We report here that in mice prophylactically administered IVIg, 34-1-2s adverse events are completely prevented and the acute lung injury at least, appeared to be prevented by IVIg's ability to restrict neutrophil ROS production.

## Results

### IVIg prophylactic treatment prevented 34-1-2s-induced hypothermia in SCID mice

**Figure 1:**
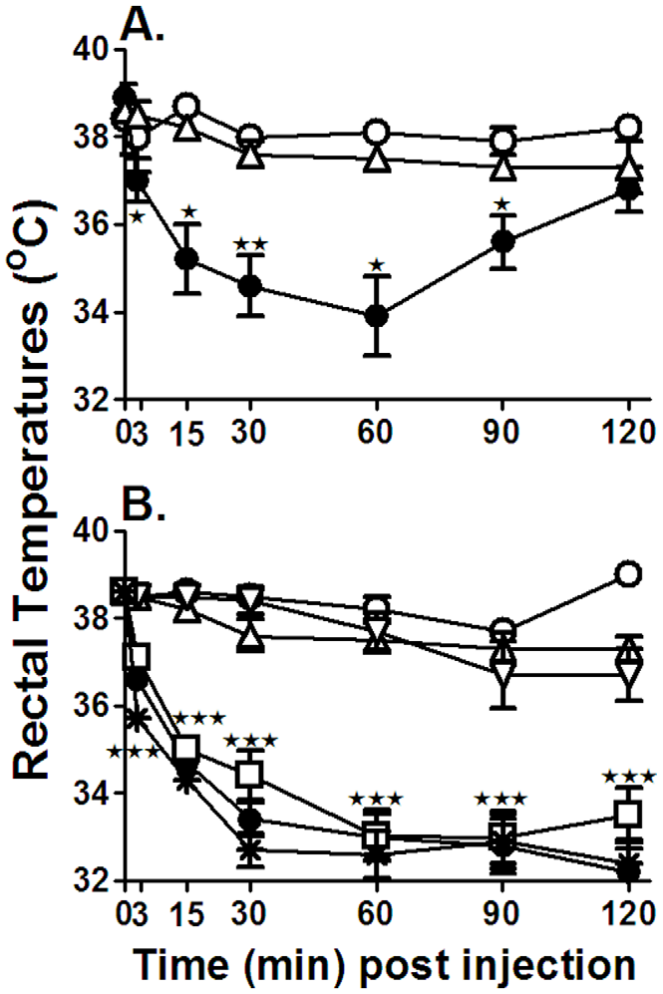
IVIg prevents 34-1-2s-induced hypothermia in BALB/c and SCID mice. Systemic shock was measured by rectal temperatures in A) BALB/c mice or B) SCID mice. In both panels, mice were either not-treated (○) or administered 34-1-2s (•, 9 mg/kg for BALB/c mice and 2 mg/kg for SCID mice iv) or treated prophylactically with 2 g/kg of IVIg 18 hours before 34-1-2s infusion (▵). Some SCID mice in panel B were also treated with 1 g/kg of IVIg 18 hours before 34-1-2s infusion (▿), 1 g/kg of IVIg within 3 minutes after 34-1-2s infusion (□) or 2 g/kg HSA (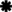). Rectal temperatures were monitored at the indicated times after TRALI induction. There were at least 5 mice per group. The data is expressed as mean ±SEM rectal temperatures. Significance was determined by Student's t test at each time point between 34-1-2s infused mice (•) and IVIg treated (▵) mice (★, p<0.02; ★★, p<0.01; ★★★ p<0.0001).

Body temperatures were monitored as a measure of systemic shock induced by the 34-1-2s infusions. Compared with control naïve mice, when either BALB/c or SCID mice were administered 9 mg/kg or 2.3 mg/kg 34-1-2s respectively, rectal temperatures began to decrease within 3 minutes post infusion and were significantly reduced by 30 minutes post infusion (Figure 1). Maximal reductions were observed by 90 min post infusion and did not recover in SCID mice whereas BALB/c mouse temperature began to recover within the 2 hour duration of the experiment (Figure 1). In contrast, compared with HSA-treated control mice, if mice were prophylactically treated with either 1 or 2 g/kg of IVIg 18 hours before 34-1-2s infusion, no significant hypothermia was observed at any time (Figure 1). To determine if IVIg could protect mice undergoing TRALI reactions, when the rectal temperatures decreased by 1°C in 34-1-2s-treated mice, they were then treated iv with 1 g/kg of IVIg. IVIg treatment of either BALB/c or SCID mice did not rescue the 34-1-2s-induced hypothermia (Figure 1).

### IVIg reversed 34-1-2s-induced edema

**Figure 2:**
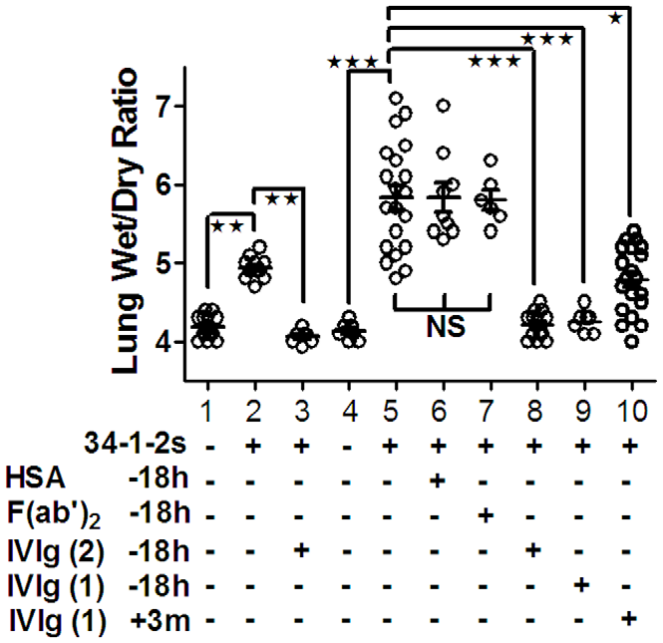
IVIg prevents 34-1-2s-induced lung damage in BALB/c and SCID mice. Wet/Dry ratios in non-treated (−) naïve BALB/c mice (column 1) and treated (+) BALB/c mice (columns 2,3) or SCID mice (columns 4–10) administered the indicated treatments (below left). TRALI was induced with an iv injection of 2 mg/kg 34-1-2s iv for SCID mice and 9 mg/kg 34-1-2s for BALB/c mice. Some mice were treated prophylactically 18 hours before TRALI induction with either 2 g/kg of IVIg (IVIg (2) −18 h), 1 g/kg of IVIg (IVIg (1) −18 h) or a control infusion with 2 g/kg human serum albumin (HSA −18 h) or 1 g/kg IVIg F(ab′)_2_ fragments (F(ab′)_2_ −18 h). The other mice were treated within 3 minutes after TRALI induction with 1 g/kg of IVIg (IVIg (1) +3 m). Two hours after TRALI induction, the mice were sacrificed and lung wet/dry (W/D) ratios were measured. The data is expressed as the individual mouse W/D ratios in each group and means±SEM are also shown. Statistics were performed using a one way ANOVA and the ★ indicates significance (p<0.001) between the indicated comparisons.

Post-mortem measurement of Wet/Dry (W/D) lung weight ratios were used to determine lung water content, an indicator of pulmonary vascular barrier failure. Compared with untreated naïve mice or mice treated with HSA, W/D lung weight ratios of SCID mice infused with 2 mg/kg 34-1-2s significantly increased by 2 hours post 34-1-2s-infusion (Figure 2). This was also true in BALB/c mice given the higher dose (9 mg/kg) of 34-1-2s, except that the magnitude of lung injury was considerably less compared with SCID mice (Figure 2). In contrast, prophylactic IVIg treatment with either 1 or 2 g/kg of IVIg 18 hrs prior to 34-1-2s infusion completely prevented this form of lung injury in either mouse strain (Figure 2). This protection was dependent on intact IVIg as F(ab′)_2_ fragments prepared from the IVIg did not affect the 34-1-2s-induced lung damage in SCID mice (Figure 2). Acute treatment with 1 g/kg IVIg within 3 minutes after 34-1-2s infusion also significantly reduced the W/D ratio in BALB/c mice and tended to reduce the ratio in the SCID mice (Figure 2).

### IVIg treatment prevented 34-1-2s-induced mortality

**Figure 3:**
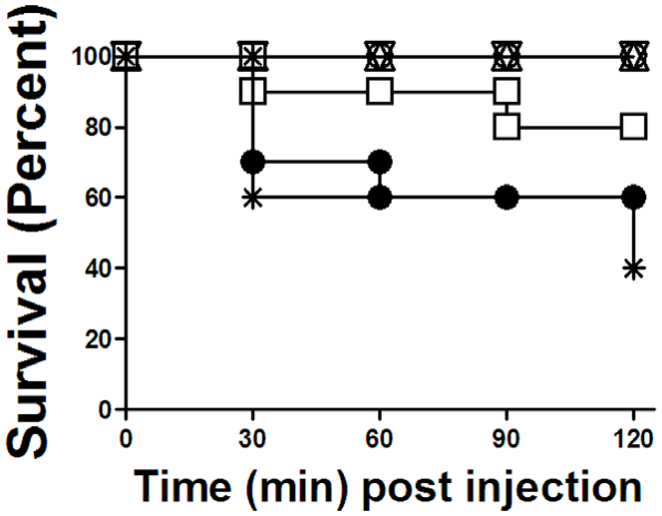
IVIg prevents 34-1-2s-induced TRALI mortality in SCID mice. Kaplan-Meier survival plots of SCID mice (N = 10 for each dose group). SCID mice were not treated (○) or administered 34-1-2s (•, 2 mg/kg). Some mice were treated prophylactically 18 hours before 34-1-2s infusion with either a control infusion of 2 g/kg HSA (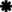) or 2 g/kg of IVIg (▵) or 1 g/kg of IVIg (▿). The other mice were treated within 3 minutes after TRALI induction with 1 g/kg of IVIg (□). The data is expressed as percentage of mice surviving at the indicated times after TRALI induction. No BALB/c mice treated with 9 mg/kg 34-1-2s died during the experimental protocol. Significance was determined by a Log ranked Mantel-Cox test between 34-1-2s infused mice (•) and prophylactically IVIg treated (▵, 2 g/kg) mice); p = 0.0289 (the survival curves are significantly different.

Compared with control non-treated mice or 34-1-2s-treated BALB/c mice, when SCID mice were infused with 34-1-2s, forty percent mortality was observed within 60 minutes post mAb infusion (Figure 3). In contrast, compared with control HAS infusions, prophylactic IVIg treatment with either the 1 or 2 g/kg doses 18 hours before TRALI induction completely prevented 34-1-2s-induced mortality (Figure 3). Similarly, acute treatment with 1 g/kg of IVIg within 3 minutes after 34-1-2s infusion also reduced mortality in the SCID mice (Figure 3).

### IVIg treatment does not prevent MIP-2 production nor pulmonary neutrophil accumulation in TRALI

**Figure 4:**
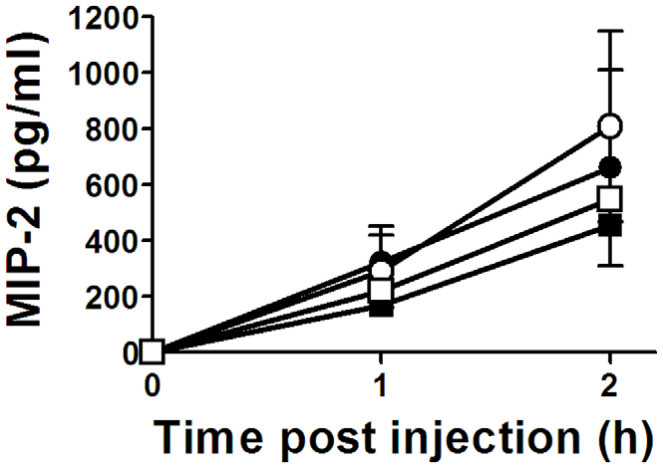
IVIg does not prevent 34-1-2s-induced MIP-2 production in vivo. BALB/c (○) or SCID mice (□) were injected iv with 2 mg/kg or 9 mg/kg 34-1-2s respectively or BALB/c (•) or SCID mice (▪) were first treated with 2 g/kg IVIg (−18 h) before 34-1-2s injection and MIP-2 levels were measured in the sera of the mice at the indicated times by commercial ELISA. The data is expressed as mean MIP-2 concentration (+SEM) from 5 mice in each group.

**Figure 5:**
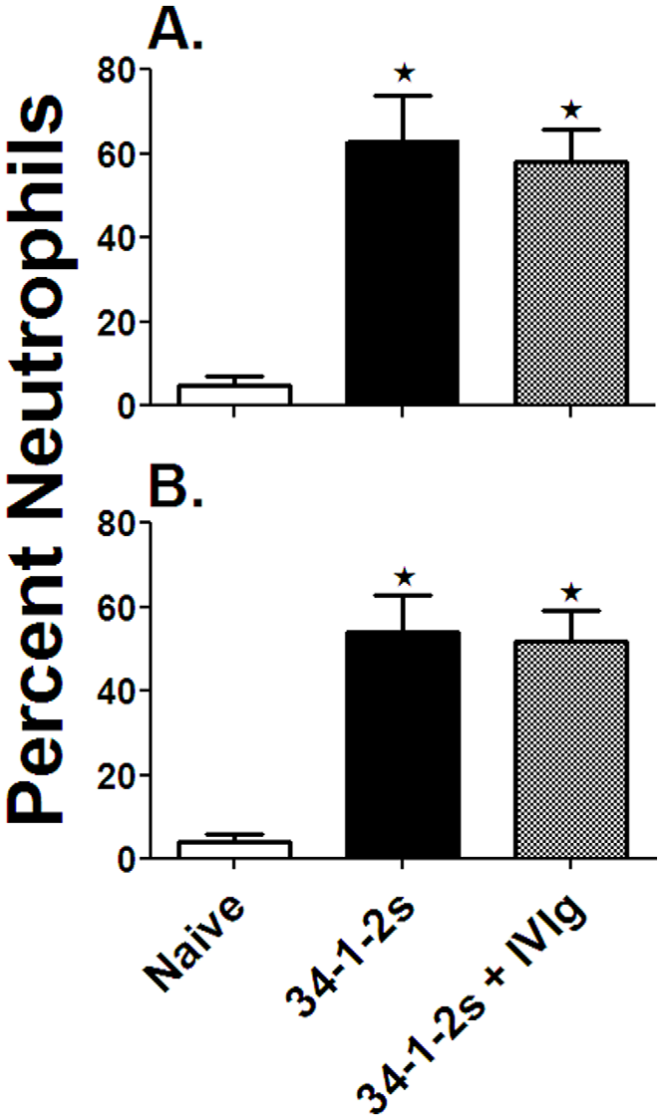
IVIg does not prevent 34-1-2s-induced pulmonary neutrophil accumulation in BALB/c or SCID mice. Percentage of neutrophils in cytospin samples of lungs from A) BALB/c mice or B) SCID mice. Mice were either non-treated (Naïve), 34-1-2s-treated (9 mg/kg for BALB/c mice and 2 mg/kg for SCID mice) or 34-1-2s and 2 g/kg IVIg treated. Neutrophils were enumerated on cytospin slides using ImageJ software. The data are expressed as percent neutrophils in total nucleated cells. The ★ indicates significance (p<0.05) between naïve mice and the other groups of mice Determined by Student's t test.

The levels of the neutrophil chemotactic (CXCL2) chemokine MIP-2 (murine equivalent to human IL-8) in serum and pulmonary neutrophils were enumerated in the mouse groups. Despite IVIg's prophylactic ability to prevent the 34-1-2s TRALI symptoms above, the IVIg pre-treatment did not prevent either in vivo 34-1-2s-induced MIP-2 production (Figure 4) nor did it reduce neutrophil accumulation within the lungs (Figure 5) of either BALB/c or SCID mice.

### IVIg effects in TRALI are associated with inhibition of 34-1-2s-induced neutrophil ROS pathway activation

**Figure 6:**
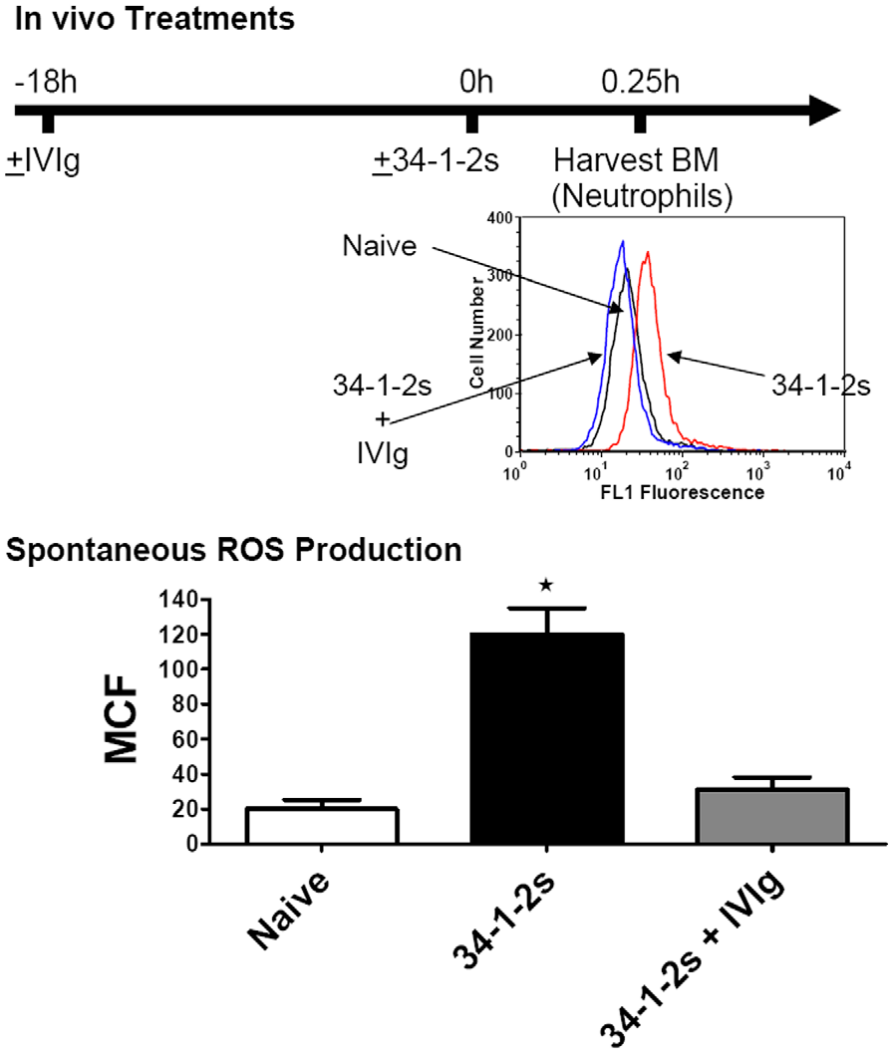
IVIg prevents spontaneous ROS production in SCID mice. The effect of the indicated in vivo treatments on spontaneous ROS production. SCID mice were either not treated (Naïve) or treated with 2 g/kg IVIg and/or 2 mg/kg 34-1-2s and their neutrophils were purified from bone marrow and tested for their ability to produce ROS spontaneously in vitro. Results are expressed as mean channel fluorescence of DHR-123 fluorescence (±SEM) from 10 mice per group. The time line at the top demonstrates the protocol timing and the insert graph shows a typical histogram analysis of spontaneous DHR-123 fluorescence in the 3 indicated mouse groups. The ★ indicates significance (p<0.05) between naïve mice and the other groups of mice determined by Student's t test.

**Figure 7:**
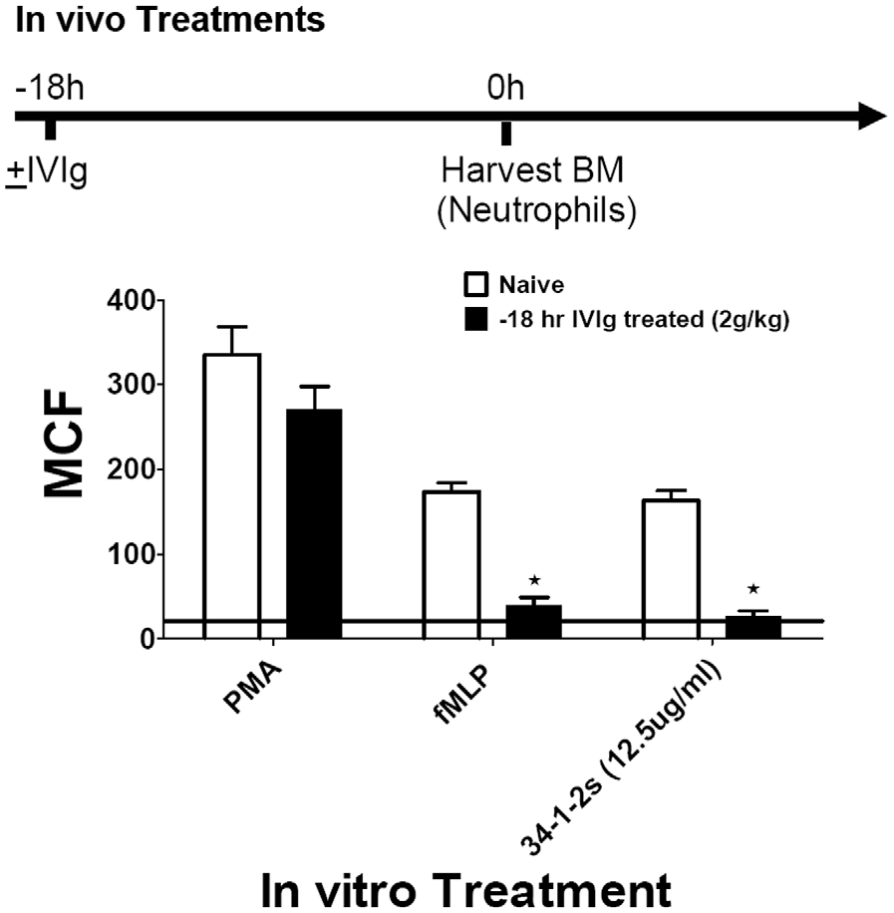
IVIg prevents in vitro stimulated ROS production in SCID mice. The effect of in vivo IVIg treatment on neutrophil ROS production stimulated in vitro. Mice were either not treated (White bars) or treated with 2 g/kg IVIg (Black bars) and their neutrophils were purified and tested for their ability to produce ROS in vitro after stimulation with 34-1-2s, fMLP or PMA. Results are expressed as mean channel fluorescence of DHR-123 fluorescence (±SEM) from 5 mice per group. The horizontal line represents baseline ROS production in non-stimulated neutrophils from naïve SCID mice. The time line at the top demonstrates the protocol timing. The ★ indicates significance (p<0.05) between non-treated and IVIg treated mice in each stimulation and determined by student's t test.

To assess the effects of IVIg on 34-1-2s-induced neutrophil ROS production, bone marrow derived neutrophils were purified from the indicated mice, labeled with DHR-123 and analyzed by flow cytometry. Neutrophils purified from 34-1-2s-treated mice produced significantly more spontaneous ROS as compared with naïve mice (Figure 6). In contrast, if the mice were pre-treated with 2 g/kg IVIg 18 hours before 34-1-2s-TRALI induction, the neutrophils maintained near basal levels of spontaneous ROS production similar to naïve mice (Figure 6). Neutrophils isolated from IVIg pretreated mice also displayed significantly decreased ROS production in response to in vitro stimulation with 34-1-2s (Figure 7). When the neutrophils from IVIg pretreated mice were assessed for other intracellular pathways leading to ROS activation, it was observed that the fMLP but not the PMA-induced pathway was inhibited by IVIg (Figure 7).

### IVIg treatment on acid-induced acute lung injury (ALI)

**Figure 8:**
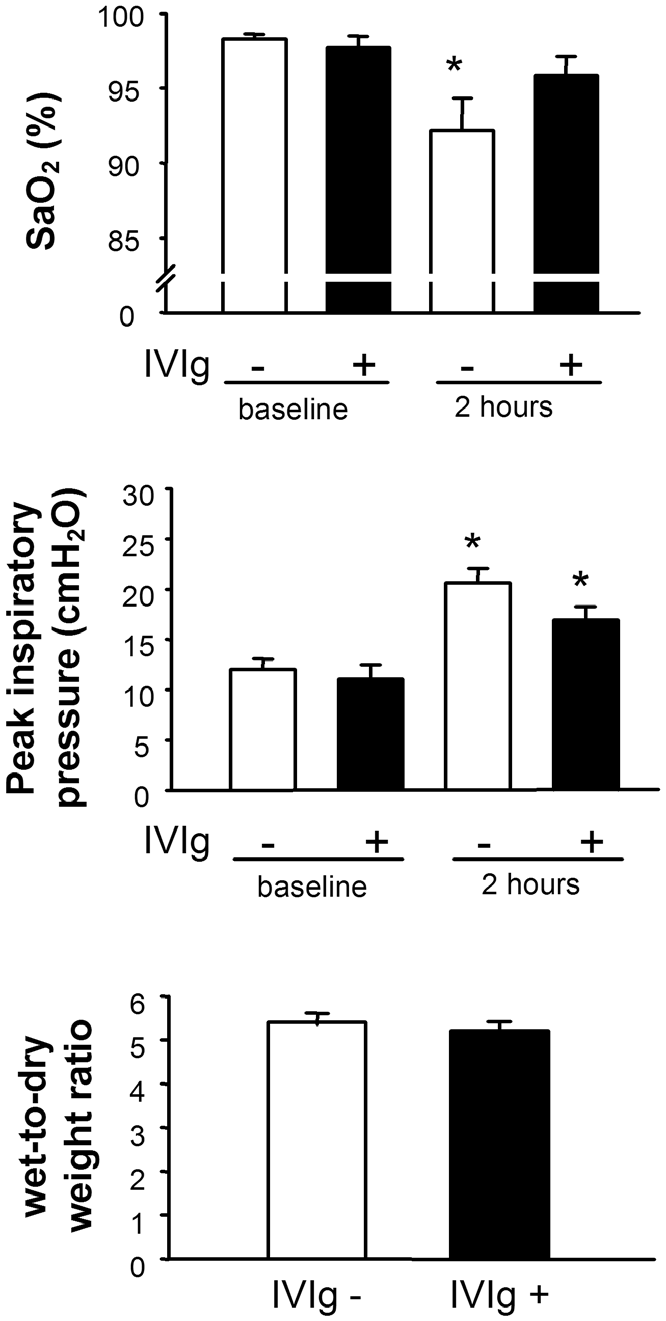
IVIg has a modest protective effect in acid-induced lung injury. IVIg partially protects from arterial hypoxemia, but not changes in lung mechanics and edema formation in acid-induced lung injury. SCID mice were pretreated with either saline (White bars) or 2 g/kg of IVIg (Black bars) 18 hours before acid induced lung injury was induced by intratracheal instillation of HCl (2 mL/kg bw at pH 1.0) (A) Arterial oxygen saturation (SaO_2_) levels and (B) peak inspiratory pressure were determined at baseline and 2 hours after instillation of HCl. Wet-to-dry lung weight ratios (C) were determined 2 h after HCl instillation. Data are expressed as means±SEM from n = 5 animals in each group. The ★ indicates significance (p<0.05) between baseline and the treatment groups determined by Student's t test.

As IVIg was able to block some pathways of neutrophil activation but not others, we examined its protective effect in a non-antibody acid induced lung injury model. Prophylactic treatment with 2 g/kg of IVIg ip 18 hours before the induction of acid-induced ALI showed a slight trend towards an attenuated injury response in that the decline in SaO_2_ subsequent to acid instillation was largely blocked, yet no significant differences in peak inspiratory pressures or mouse W/D lung weight ratios were detectable between saline and IVIg pretreated mice (Figure 8).

## Discussion

The 34-1-2s murine acute lung injury model has been shown to be a good approximation of human TRALI in that recipient mice exhibit increased pulmonary permeability, pulmonary pathology and mortality which are mediated by activated pulmonary neutrophils in an FcR-dependent manner [20]. There is also evidence suggesting that 34-1-2s can activate the generation of C5a which stimulates macrophage ROS production [22]. In our hands, BALB/c mice have milder respiratory symptoms after 34-1-2s infusion whereas SCID mice are extremely hypersensitive to 34-1-2s [21]. Thus BALB/c mice may represent the milder form of TRALI termed transfusion associated dyspnea (TAD) whereas SCID mice share more in common with the severe, fatal form of TRALI. We report here that prophylactic IVIg administration completely prevented TAD and the severe form of TRALI e.g. hypothermia, lung damage and mortality. The TRALI protective effect of IVIg was not due to reducing pulmonary neutrophil accumulation but was associated with direct inhibition of 34-1-2s's ability to activate ROS production. These results suggest that IVIg may have a beneficial effect in reversing and preventing severe TRALI reactions.

Although there are now at least 4 case reports (Evidence level III) that have shown that IVIg infusions may be associated with TRALI reactions [40]–[43], we reasoned that given the enormous amount of IVIg infused into many thousands of patients world-wide, these IVIg-TRALI adverse events actually represent extremely rare occurrences. Of interest, there are also several clinical and experimental reports that have demonstrated an IVIg-beneficial effect in other types of inflammatory lung injury. For example, IVIg infusion reduces lung injury in patients with Adult-onset Still's disease, patients after cardiac surgery as well as a variety of patient groups that have an infection-related lung injury [44]–[48]. We hypothesized that since IVIg treatment has beneficial effects in a wide variety of antibody-mediated and inflammatory pathologies, it would reduce the adverse effects of antibody-mediated TRALI.

Experimentally, prophylactic treatment of mice with 1 or 2 g/kg of IVIg 18 hours before TRALI induction completely prevented systemic shock, lung damage and mortality induced by the 34-1-2s antibody (Figures 1,2,3). While this may not be logistically feasible clinically, it demonstrates at least, that the anti-inflammatory effects induced by IVIg may be sufficient to completely prevent antibody-mediated TRALI effects. To determine if acute TRALI reactions could be modulated by IVIg, we first administered 34-1-2s and upon detecting a 1°C rectal temperature fall (within 3 minutes after 34-1-2s infusion), the mice were infused iv with 1 g/kg of IVIg. We observed that while TRALI reactions still occurred particularly related to reduced rectal temperature, the severe TRALI reactions e.g. lung edema and mortality were reduced. The apparent reduced protection of the acute IVIg treatment may be due to the smaller dose of IVIg that was given by the iv route because of volume restrictions. Nonetheless, IVIg was able to acutely reduce the severe effects of 34-1-2s supporting the notion that IVIg could be used as a therapy for transfusion recipients undergoing severe TRALI reactions.

Of interest, IVIg's protective effects against lung damage were Fc-dependent as F(ab′)_2_ fragments prepared from the IVIg were not effective in reversing the lung damage. These results suggest that the mechanism of action of IVIg in reversing TRALI may have similarities to the Fc-dependent beneficial effects of IVIg in other antibody-mediated disorders such as immune thrombocytopenia (ITP). Many experimentally supported hypotheses of IVIg's mechanism of action in ITP have been proposed including, for example, reticuloendothelial Fc receptor blockade [30], Fc gamma inhibitory receptor (R) mediated inhibition of phagocytosis [31], antiidiotypic regulation [32], [33], cytokine alterations [34], [35] and IVIg-mediated dendritic cell suppression of inflammation [36]. More recently, elegant studies have demonstrated that IVIg may also mediate its effects by modulation of pro-inflammatory cytokines such as IL-17 and anti-inflammatory T regulatory cells which may exert their immunosuppressive effects via inhibition of dendritic cells [37]–[39]. It is possible that IVIg may utilize one or more of these mechanisms to prevent antibody-mediated TRALI reactions. We are currently studying this.

Approximately 4% of surgical patients undergoing a cardiopulmonary bypass (CPB) procedure have acute lung injury episodes and have an increased risk of death [49] ^and^ the majority of these patients are transfused [50]. Recently, a large prospective case controlled clinical study of cardiac surgery patients showed a high incidence (approximately 2.4%) of TRALI despite the implementation of male donor plasma [14]. It suggested a need for additional interventions in the susceptible population as has also been previously postulated [51], [52]. It is plausible that these large elective patient groups could benefit from prophylactic IVIg therapy before the procedure.

IVIG's effects were not simply due to neutralization of 34-1-2′s because binding of 34-1-2s to leukocytes as measured by flow cytometry was not inhibited by titrations of IVIg in vitro (JWS, unpublished, Feb, 2011) and in vivo, IVIg failed to reduce 34-1-2s-induced serum MIP-2 levels (Fig. 4) which are dependent on the antibody's ability to bind to its cognate antigen MHC class I. How IVIg mediates the reversal of TRALI is currently unknown but there are clues that may shed light on its mechanism of action. For example, there is evidence that IVIg can affect neutrophil adhesion to endothelial cells and neutrophil apoptosis in an Fc dependent manner [53]–[55]. It is also possible that IVIg has the ability to dampen neutrophil activation in the lung and rescues the adverse pulmonary effects. We tested this and found that although prophylactic IVIg treatment did not significantly affect the ability of 34-1-2s to stimulate MIP-2 production (Fig. 4) nor cause pulmonary neutrophil accumulation (Fig. 5), it was able to significantly inhibit 34-1-2s's ability to induce neutrophil ROS production in vitro (Fig. 6). In contrast, IVIg was not effective in affecting PMA-induced neutrophil ROS production. Although the relationship between acute lung injury and protein kinase C activation is complex and has not yet been completely defined [56], these pathways of lung injury are fundamentally different with IVIg only being effective in a particular pathway of neutrophil activation. Collectively, these results suggest that IVIg mediates its effects via an FcR-dependent anti-inflammatory effect on neutrophils that limits ROS production rather than the prevention of binding of 34-1-2s to it's cognate antigen e.g. MIP-2 production or other necessary steps necessary for the chemoattraction and physical trapping of neutrophils in the lung.

The effects of IVIg appeared to be rather specific to TRALI because IVIg had only modest protective effects on acid-induced ALI (Figure 7). Indeed, IVIg largely prevented acid-induced hypoxemia, yet it did not attenuate the increase in peak inspiratory pressure indicating reduced lung compliance; this correlated with no reduction in the W/D lung weight ratio. While the partially beneficial effects of IVIg in non-transfusion related ALI deserve further analysis in future studies, the present observations support the notion that IVIg confers its beneficial actions via distinct anti-inflammatory pathways specific to the pathophysiology of the disease being treated. In particular, IVIg was effective in 34-1-2s-induced neutrophil activation and TRALI.

In summary, we have demonstrated that in contrast to the few reports showing that IVIg may be associated with TRALI reactions, prophylactic IVIg administration significantly and substantially protects mice from antibody-mediated TRALI and does so at the level of neutrophil activation. Thus, IVIg could be considered as a potential therapeutic for patients predisposed to TRALI reactions.

## Methods

### Mice

Male BALB/c mice or CB.17 (H-2^d^, CB17/Icr-*Prkdc^scid^*/IcrCrl) severe combined immunodeficient (SCID) mice, 6–12 weeks of age, were obtained from Jackson Laboratories (Bar Harbor, ME) or Charles River Laboratories (Montreal, PQ, Canada). All animal studies were approved by the St. Michael's Hospital Animal Care Committee (ACC Protocol#108). To address IVIg's effects in the absence of endogenous murine IgG, SCID mice were tested for serum IgG by a murine IgG ELISA (Cedarlane Laboratories, Mississauga, ON) and any leaky mouse (>30 ug IgG/ml serum) was excluded from study.

### IVIg administration

Gamunex® (10%, Telecris Biotherapeutics, Mississauga, ON) was used as IVIg in all experiments. For prophylactic treatment, mice received doses (1 or 2 g/kg) of IVIg intraperitoneally (ip) 18 hours before TRALI induction with 34-1-2s. These ip doses were well tolerated in control mice. To attempt a more clinically relevant treatment scenario, some mice were treated intravenously (iv) with a 1 g/kg dose of IVIg after the first symptom of 34-1-2s-induced TRALI was observed (a rectal temperature decrease of 1°C within approximately 3 minutes post 34-1-2s injection). The 1 g/kg dose chosen was the highest iv (approximately 200 uL) dose tolerated by control mice without causing volume overload symptoms (lung edema).

Control human serum albumin (HSA, 25% w/v, Baxter Healthcare Corporation, Westlake Village, CA, USA) was diluted to a final concentration of 10% (w/v) with sterile PBS (pH 7.4) and administered ip at a dose of 2 g/kg. Although the albumin dose has a greater oncotic load than does IVIg, it did not adversely affect the mice.

### 34-1-2s and Antibody-mediated TRALI Induction

The hybridoma 34-1-2s (ATCC, Manassas, VA) produces a monoclonal antibody (IgG_2a_, κ) against H-2K^d^ and H-2D^d^ MHC class I molecules [57]. It was grown in protein-free hybridoma medium, PFHM II, (Invitrogen, Burlington, ON) in CELLine flasks (BD Biosciences, Bedford, MA) at 37°C and 5% CO_2_.

For antibody-mediated TRALI induction, mice were weighed and then challenged intravenously (iv) via tail vein injection with 34-1-2s; SCID mice were treated with 2 mg/kg 34-1-2s (approximately 50 ug of antibody in a 100 ul infusion per mouse) and BALB/c mice were treated with 9 mg/kg 34-1-2s (approximately 200 ug of antibody in a 100 ul infusion per mouse); these infusions approximate the volume of a plasma transfusion in humans. The indicated physiological measurements were then performed at the indicated time points post 34-1-2s infusion.

### Acid-induced Acute Lung Injury (ALI) Induction

To compare if IVIg affects a more direct form of acute lung injury, acid induced lung injury was also performed. Briefly, SCID mice were anesthetized by intraperitoneal injection of medetomidine (0.5 mg/kg, Domitor; Dr E. Graeub AG), fentanyl (0.05 mg/kg; Janssen Cilag), and midazolam (5 mg/kg, Dormicum; Roche) and placed on a homeothermic blanket (Harvard Apparatus) in supine position. Body temperature was maintained at 37°C with a feedback coupled rectal thermoprobe. After tracheotomy, mice were intubated with a polyethylene tube (Portex FineBore Polythene Tubing, 0.58 mm ID/0.96 mm OD; Smiths Medical International) and ventilated with room air at 100 breaths/minute (tidal volume of 10 mL/kg at a positive end-expiratory pressure of 1 cmH_2_O; miniVent, Harvard Apparatus). Airway pressure was digitally recorded (DASYlab 32; Datalog GmbH) and arterial oxygen saturation (SaO_2_) was continuously monitored by pulse oximetry (MouseOx; Starr Life Sciences). Following baseline recordings, 2 mL/kg of hydrochloric acid (pH 1.0) was instilled into the trachea, and arterial oxygen saturation and airway pressure were monitored for 2 hours at which time mice were sacrificed and lung wet-to-dry weight ratio was quantified as a measure of lung edema.

### Body Temperature measurements

Rectal temperatures were measured as an indicator of systemic shock at 30 minute intervals up to 120 minutes post 34-1-2s mAb infusion using a RET-3, Rectal probe for mice (VWR International, Mississauga, ON) connected to a Traceable Digital Thermometer (Model 77776-726, Physitemp Instruments, Inc., Clifton, NJ).

### Wet/Dry Lung Weight Ratio

As a measure of pulmonary edema, wet-to-dry lung weight ratios were determined as previously described [21]. At the indicated time post 34-1-2s infusion, mice were anaesthetized using Avertin ip (2% final in PBS) and the chest cavity was exposed. The left lung was excised from each mouse, weighed (wet weight) and then dried in an oven at 60°C for ≥48 hrs and then re-weighed for dry weight. The wet-to-dry weight ratio was calculated as: net wet weight/net dry weight.

### MIP-2 Measurements

Blood was collected from the indicated mice and sera was generated on ice and tested for the presence of the CXCL2 chemokine Macrophage Inflammatory Protein 2 (MIP-2) using an ultra-sensitive commercial solid-phase ELISA kit (Mouse CXCL2/MIP-2 Quantikine ELISA Kit, R&D Systems, Cedarlane Laboratories, Burlington, ON, Cat# MM200). The MIP-2 assay had a sensitivity of >1.5 pg/ml.

### Pulmonary neutrophil accumulation

Pulmonary neutrophils were enumerated as previously described [21]. Briefly, each mouse was anaesthetized using Avertin ip (2% final in PBS) and the chest cavity was exposed and the right lung was isolated and excised. The lung was homogenized and the cell suspension produced was filtered through a 40 µm cell strainer (BD Biosciences, Bedford, MA). The cell suspension was then washed and red blood cells were removed by Ammonium Chloride/Potassium (ACK) lysis solution (0.15 M NH_4_Cl, 10 mM KHCO_3_, Na_2_EDTA, pH 7.2–7.4). The cell preparation was then washed twice with cold PBS and cells were mounted on microscope slides using a Shandon Cytospin 4 (ThermoFisher Scientific, Nepean, ON) and stained with a haematoxylin-and-eosin (H&E) kit (Harleco - Hemacolor, EMD Chemicals, Inc., Darmstadt, Germany). The slides were then examined by light microscopy using a microscope (Olympus Canada, Markham, ON) with a 60× oil immersion lens. PMN were enumerated by counting total nucleated cells in at least 3 equivalent fields and neutrophils were enumerated by marking with ImageJ software. Neutrophil enumeration was calculated as percent neutrophils of total nucleated cells by the formula: Neutrophil counts/Total nucleated cell counts ×100.

### Neutrophil purification and in vitro activation studies

Neutrophils from the different mouse groups were collected from the bone marrow and purified. Pulmonary neutrophils in 34-1-2s-treated mice are very difficult to separate from the inflamed lung tissue and were not used. Briefly, mice were sacrificed and their femurs were removed, cleaned of tissue, epiphysises removed and bone marrow was flushed out with a 25 g needle using an ice cold 0.1% FCS/PBS solution. The cells were filtered through a 40 um cell strainer, counted and neutrophils were purified using a mouse neutrophil enrichment kit (Stem Cell Technologies, Vancouver, CA) and a magnetic activated cell sorter as described in the manufacturers instructions. Purity of the neutrophils was >96%. For in vitro activation, 2×10^5^ purified neutrophils in 100 ul of PBS were incubated in 1 uM of the ROS-specific fluorochrome Dihydrorhodamine 123 (DHR-123) for 10 minutes at 37°C. The labeled neutrophils were then incubated with either nothing (spontaneous ROS), 2 ug/ml phorbol 12-myristate 13-acetate (PMA) or 100 nM formyl-methionyl-leucyl-phenylalanine (fMLP) or serial dilutions of 34-1-2s for 10 minutes at 37°C. The cells were then incubated with 200 ul of DNA staining solution (LDS751) on ice for 10 minutes in dark and acquired on a FACSort flow cytometer (BD, Missisauga, ON) gated on LDS751 staining. DHR-123 fluorescence was analyzed as a measure of ROS production.

### Statistical Analysis

Significance between mouse groups was determined by various statistical methods as indicated in each figure legend.
